# Triage by cervical length sonographic measurements for targeted therapy in threatened preterm labor: A double blind randomized clinical trial

**Published:** 2017-11

**Authors:** Homeira Vafaei, Neda Rahimirad, Seyedeh Marjan Hosseini, Maryam Kasraeian, Nasrin Asadi, Hadi Raeisi Shahraki, Khadijeh Bazrafshan

**Affiliations:** 1 *Maternal-Fetal Medicine Research Center, Perinatology Ward, Shiraz University of Medical Sciences, Shiraz, Iran.*; 2 *Department of Obstetrics and Gynecology, Shiraz University of Medical Sciences, Shiraz, Iran.*; 3 *Department of Biostatistics, School of Medicine, Shiraz University of Medical Sciences, Shiraz, Iran.*; 4 *Maternal-Fetal Medicine Research Center, Shiraz University of Medical Sciences, Shiraz, Iran.*

**Keywords:** Cervical length, Preterm labor, Tocolysis, Ultrasonography.

## Abstract

**Background:**

Preterm labor and birth are associated with several neonatal complications including respiratory distress syndrome and intraventricular hemorrhage. Differentiating true and false labor pain is a dilemma to obstetricians.

**Objective:**

To elucidate the role of cervical length measurement in prediction of birth in pregnant women with threatened preterm labor.

**Materials and Methods:**

In this double blind randomized clinical trial, 120 women with gestational age <34 wk who presented painful uterine contractions randomly assigned to undergo measurement of cervical length. Patients were registered in the hospital and a unit number was given. Based on the unit numbers, patients were randomly assigned to two groups using a computerized random digit generator. All participants were managed accordingly (n=65) or to receive tocolysis as planned (n=55). Tocolysis was prescribed when cervical length was <15 mm while those with cervical length ≥15 mm were managed expectantly. Delivery within 7 days of the presentation was the primary outcome.

**Results:**

This RCT showed in case group, 78.9% of patient with cervical length <15 mm were delivered within 7 days and only 21.1% of them maintained their pregnancy. Of those with cervical length ≥15 mm, only 15.2% were delivered within the study period and the rest (84.8%) maintained their pregnancy (p<0.001).

**Conclusion:**

“Our results indicate that in women who presented preterm labor symptoms, cervical length measurement will result in decreased unnecessary tocolytic treatment. Women with cervical length ≥15mm should not receive tocolysis, however, withholding corticosteroid therapy in these patients needs further evidence.

## Introduction

Preterm labor defined as occurrence of uterine contractions accompanied by cervical changes (dilation and effacement) before 37 wk of gestation is considered ,estimated prevalence of preterm birth is 5-10% of all pregnancies (1). Preterm birth is increased short term complications ascribed to immaturity of multiple organ systems &long term adverse effects on health in whole life (2). Vasodilatory effects of progesterone on the vessels of fetus and uterus, seems be effective in the prevention of preterm birth (3). 

However, the drug of choice is yet to be identified because of complications associated with most of them (3, 4). Several criteria have been introduced for diagnosis of preterm labor. According to Creasy, preterm labor is defined as uterine contractions (>4 contractions per 20 min), cervical dilation 2≥ cm in a nulliparous and 3≥ cm in a multipara) and cervical effacement (>80%) or uterine contractions and cervical change (5). However, preterm labor is usually over-diagnosed and unnecessary tocolytic and corticosteroid treatment are administered (6). Several other methods have been used for diagnosis of preterm labor including transvaginal ultrasonography. Measurement of cervical length using transvaginal ultrasonography has been shown to predict preterm birth in those presenting with threatened preterm labor (7, 8). A systematic review done in 2008 showed cervical length measurement using transvaginal ultrasonography has high accuracy in predicting spontaneous preterm birth in high risk asymptomatic population (9).

Alfirevic and his coworkers found that those women with threatened preterm labor who has cervical length more than 15 mm should not receive any tocolysis because the preterm birth will not occur in these patients (10). In another study by Tsoi and his colleagues, 99% of women with cervical length ≥15 mm did not deliver within 7 days without receiving tocolytic therapy (11). 

According to these observations and in order to elucidate the role of cervical length measurement in prediction of preterm birth in those with threatened preterm labor, we performed this randomized clinical trial.

## Materials and methods

This randomized clinical trial was performed in Hafez hospital, Shiraz University of Medical Sciences, Shiraz, Iran during November 2010 to February 2015. 

All women with live, singleton pregnancies who presented true uterine contractions before 34 wk of gestation age (at least 4 contractions in 20 min interval) that were selected to receive tocolytic and steroid therapy were included. Cervical effacement was not considered as a criterion for inclusion or exclusion in this trial. Women with cervical dilatation >5 cm, polyhydramnios (amniotic fluid index greater than 24 cm), macrosomia (estimated fetal weight >90^th^ centile), multiple pregnancy, suspected intrauterine infection, rupture of membranes, vaginal bleeding (more than bloody show), major medical disorders in mother, contraindicated for vaginal delivery (e.g. placenta previa), non-reassuring fetal heart monitoring, fetal anomaly, any contraindication for administer of beta-sympathomimetic drugs and previous treatment with tocolytics/corticosteroid in this pregnancy were excluded. No other tests including fibronectin were used for diagnosis of preterm labor. Transvaginal sonography for cervical length measurement was done by an expert obstetrician.”

**Study protocol**

Patients were selected by labor residents at the time of admission. A thorough history and physical examination was performed by the residents including the digital vaginal examination. The findings including the fetal presentation, cervical dilatation, effacement and past medical history were recorded in the structured questionnaire. A total of 120 patients with preterm labor were enrolled in this study being randomized to two groups including: 65 patients undergoing cervical length measurement by transvaginal ultrasonography for further management (case group) and 55 patients who received tocolytic and corticosteroid therapy (control group).

Those who were assigned to the case group underwent transvaginal ultrasonography for measuring the cervical length by one expert obstetrician. Neither residents nor sonographers were not aware of this clinical trial. Those with cervical length <15 mm were received tocolytic and corticosteroid therapy according to the hospital protocol. Expectantly management was performed when cervical length was ≥15 mm, but with persistent uterine contractions more than 4 hr, another cervical measurement was performed by the same operator. If the cervical length was become <15 mm tocolytic and antenatal corticosteroids were given.

Patients were followed for 7 days after enrollment in the study and the uterine contractions were screened. If the pain and uterine contractions were resolved, the patient was discharged and followed by the phone. All the participants were asked to record their uterine contraction in a diary manner. All the patients who had not delivered within the first 7 days after enrollment in the study, underwent uterine contraction check-up. The primary outcome of study was proportion of the patients who maintained their pregnancy at least 7 days after randomization. All the patients were observed precisely in the labor suite.

**Ethical consideration**

The study protocol was approved by the institutional review board and Ethics Committee of Shiraz University of Medical Sciences, Shiraz, Iran (Ct-88-5026, 1388-12-27). All the participants patients in case & control groups gave their informed written consent.

**Statistical analysis**

Descriptive statistics were reported as mean±SD for quantitative or frequency (%) for qualitative variables. Association between two categorical variables was assessed using chi-square or Fisher's exact test where appropriate. Normality of all the quantitative variables was approved in both groups via Shapiro-Wilk test (P>0.05 in all variables) therefore independent t-test was used to examine the difference between two groups.

All the statistical analysis performed in SPSS (Statistical Package for the Social Sciences, version 18.0, SPSS Inc, Chicago, Illinois, USA) and p<0.05 was considered significant.

**Figure 1:**
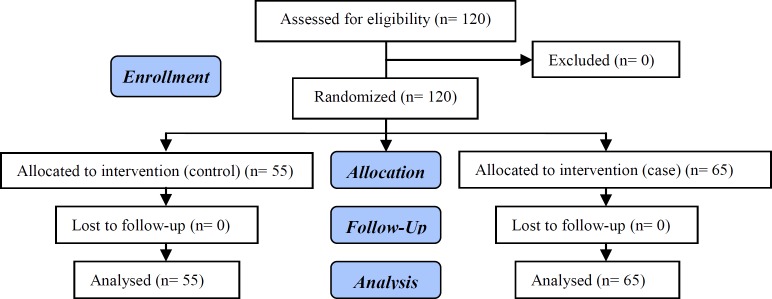
CONSORT flow diagram of the participants

## Results

The mean age of the participants was 28.4±4.3 (range 18-36 yrs.). The mean age of the patients and controls was found to be 24.8±4.6 and 24.81±4.1 yrs. respectively (p=0.978). Table I compares the demographic characteristics of cases and controls. The cervical dilation was significantly higher in the controls compared to the case group (p=0.003). While the control group had significantly higher effacement compared to case group (50.91±22.7% vs. 59.61±13.6%; p=0.015). 

Table II summarizes the pregnancy characteristics of two study groups. Cervical length in all of the 65 patients in case group was measured with a cut-off value of 15 mm. The mean cervical length in the cases was 21.4±7.9 mm (range 8-37 mm). 46 patients (70.7% of case group) had cervical length ≥15 mm, and only 19 patients (29.3% of case group) had cervical length <15 mm and received tocolytic therapy. 

Among 46 patients in case group with cervical length ≥15 mm, the uterine contractions persisted in 6 patient (13%) and cervical length measurement was repeated for them after 4 hr. In these 6 patients, mean cervical length was 20.6±5.4 mm (range 16-28 mm). The mean cervical length was decreased significantly in those with persistent contraction (p=0.008); however, none of them had cervical length <l5 mm and thus did not receive therapy. 

Of those 19 (29.3%) patients who had cervical length <l5mm, 15 (78.9%) delivered during the study period and only 4 (21.1%) maintained their pregnancy. From 46 patients with cervical length ≥15 mm, only 7 (15.2%) delivered within the study period and the rest (84.8%) maintained their pregnancy. The difference was significant at the level of p<0.001. Overall, 22 patients (0.33%) in case group delivered during the study period, compared to 13 patients (28%) in control group, the difference was not significant (p=0.31). 

**Table I:** Baseline characteristics of patients in two study groups

**Variables**		**Case group(n=65)**	**Control group (n=45)**	**p-value**
Age (yr)*		24.8 ± 4.6	24.8 ± 4.1	0.978†
BMI(Kg/m²)*		25.7 ± 2.4	25.3 ± 2.4	0.324†
Gravidity**				
	Prim gravid	24 (36.9)	26 (47.3)	0.687††
	Multigravid	41 (63)	29 (52)	
Parity**				
	Nulliparous	32 (49.2)	32 (58.2)	0.608††
	Multiparous	33 (50.8)	23 (41.8)	
Previous history of abortion**				
	No abortion	49 (75.4)	43 (78.2)	0.928††
	One abortion	13 (20.0)	10 (18.2)	
	Two abortion	3 (4.6)	2 (3.6)	
Previous history of stillbirth**				
	No stillbirth	59 (90.8)	53 (96.4)	0.198††
	One still birth	6 (9.2)	2 (3.6)	

BMI: Body mass index

* Data presented as mean±SD;

** Data presented as n (%).

† Independent *t*-test

†† Chi-square test

**Table II:** Pregnancy characteristics in two study groups

	**Case group (n= 65)**	**Control group(n= 55)**	**p-value**
Gestational age by LMP (wk)*	30.7 ± 1.6	30.9 ± 1.9	0.586
Gestational age by sonography (wk)*	30.9 ± 1.6	30.9 ± 1.9	0.901
Cervical dilatation (Cm)*	1.6 ± 0.7	1.9 ± 0.5	0.003
Cervical effacement (%)*	50.91 ± 22.7	59.61 ± 13.6	0.015
delivery during the study period**	22 (33)	13 (28)	0.31

**Independent** ***t*** **-test **

* Data presented as mean±SD;

** Data presented as n (%).

**Table III:** Relation of cervical length and duration of pregnancy

**Cervical length**	**Total**	**Delivery within 7days**	**Maintaining pregnancy**	**p-value**
≥15 mm	46 (70.7)	7 (15.2)	39 (84.8)	<0.001
<15 mm	19 (29.3)	15 (78.9)	4 (21.1)	

Data presented as n (%)

Chi-square test

## Discussion

We found that approximately 80% of patients who has cervical length <l5 mm will deliver within a 7-day period despite receiving tocolytic therapy and this means that preterm birth is inevitable when the cervical canal is less than l5 mm in length. So, prescribing tocolytic agents and corticosteroids to this group seems to be reasonable and will provide the patient with a favorable result of neonate lung maturity and decreased prevalence of respiratory distress syndrome.

In another group, those with cervical length ≥15 mm, 85% maintained their pregnancy without any need for tocolytic and corticosteroid therapy, thus avoiding unnecessary treatment. These results are in agreement with several previous studies that have shown the beneficial advantages of cervical length measurement in threatened preterm labor and eligibility of selecting the patient for treatment with tocolytic agent and corticosteroids (10, 11).

In this regards, Alfirevic and coworkers performed a randomized clinical trial in order to test the supposition of managing threatened preterm labor based on measuring of cervical length by ultrasonography can decrease the number of women who receive inadvisable treatment. They included 41 patients with threatened preterm labor who were assigned to receive tocolytic and corticosteroid therapy. They divided their patients into two groups to undergo cervical length measurement (n=21) or to receive conventional treatment (n=20). The treatment was avoided in 14 women who had cervical length ≥15 mm while the other 7 patients received tocolytic and corticosteroids. The prescription of corticosteroids was significantly higher in control group compared to ultrasonography group (14% vs. 90%; relative risk (RR) 0.16; 95% CI, 0.05-0.39). Prevention of tocolytic therapy was also significant between two groups (33.3% vs. 100%; RR 0.3; 95% CI, 0.15-0.54). None of the neonates were born without receiving a full course of corticosteroids (10).

In another study by Tsoi and coworkers, the efficacy of cervical length measurement for distinguishing between true and false labor was examined. They included 216 singleton pregnancies at 24-36 (mean, 32) wk of gestation referring with threatened preterm labor. Delivery within 7 days of the presentation was considered as the primary outcome. Of 173 patients with cervical length ≥15mm, only 1 delivered within the 7-dayperiod. Of 43 patients with a cervical length <l5mm, 16 delivered within the 7 days of which 6/14 (42%) were treated with tocolytics and 10/29 (35%) were managed expectantly. They showed that cervical length was the only predictor of preterm birth (OR=101, 95% CI 12-800, p<0.0001) independent of other demographic factors including ethnicity, parity, maternal age, gestational age, body mass index, history of previous preterm delivery, smoking, frequency of contractions or use of any tocolytics (11).

Fuchs and colleagues also tried to investigate the role of cervical length measurement in differentiating false and true labor pains they included 253 singleton pregnancies presenting with painful uterine contractions before 34 wk of gestation. Cervical length was measured and the cut-off of 15mm was considered the reference for prescribing treatment. Delivery within 7 days of the presentation was considered the main outcome. 21/253 (8.3%) delivered within the study period was reversely correlated with cervical length. Only 4 (1.8%) of 217 patients with cervical length ≥15 mm delivered within 7 days while 47.2% of those with a cervical length <l5 mm delivered within 7 days’ despite receiving tocolytic therapy. The delivery in threatened preterm labor patients was attributed to cervical length, frequency of contractions at presentation, history of previous preterm delivery and vaginal bleeding. It was not found to have correlation with ethnicity, maternal age, parity, smoking or using tocolysis, antibiotics or steroids. It is corroborated by univariate as well as multivariate analyses that cervical length is a significant independent predictor of delivery within 7 days in this population (12).

All these studies, like ours, show that measuring cervical length in those presenting with threatened preterm labor is a powerful tool for differentiating false and true labor pains and using this modality will decrease the unnecessary treatment. However, a question rises here and that is whether this approach will result in widespread of transvaginal ultrasonography. Withholding corticosteroid therapy in those with cervical length ≥15 mm will also result in babies born with inadequate corticosteroid therapy. However, it was shown that this risk is unlikely to be greater than 2-3% (14). 

Prospective cohort following patients till birth is needed in order to clarify this issue. In conclusion, measurement of cervical length in those with threatened preterm labor will result in reducing inappropriate tocolytic treatment. Although in women with cervical length ≥15 mm should not administer tocolysis, the prevention of using corticosteroid therapy in these patients needs further evidence.

## Note

***Registration ID in IRCT:*** ***IRCT2017092317034N5***
